# High fatigue levels among psychiatric outpatients – the validity of the Danish Patient Reported Outcomes Measurement Information System Fatigue Short-Form (PROMISF-SF)

**DOI:** 10.1186/s41687-025-00837-w

**Published:** 2025-01-21

**Authors:** Ragnar Klein Olsen, Sidse M. Arnfred, Christina Madsen, Oliver Rumle Hovmand

**Affiliations:** 1 Department of Clinical Medicine, Faculty of Health, University of Copenhagen, Copenhagen, Denmark; 2 Psychiatry West, Region Zealand Mental Health Services, Slagelse, Denmark; 3 Psychiatry South, Region Zealand Mental Health Services, Slagelse, Denmark; 4 Psychiatric Research Unit, Region Zealand Mental Health Services, Slagelse, Denmark

**Keywords:** PROMIS-F-SF, Fatigue, Anxiety, Depression, Personality disorders

## Abstract

**Background:**

Patient Reported Outcomes Measurement Information System Fatigue Short-Form (PROMIS-F-SF) is a self-administered, patient reported outcome (PRO) designed to assess fatigue in healthy and clinical populations and for tracking progress during treatment for disorders complicated with fatigue.

**Methods:**

Patients in the Mental Health Service Outpatient Clinics and healthy volunteers were invited to complete a survey, which included the Danish translation of the PROMIS-F-SF, the Chalder Fatigue Scale (CFS-11), and measures of depression and anxiety. We conducted a confirmatory factor analysis of the previously suggested single-factor structure of the instrument. We furthermore evaluated the construct validity of the PROMIS-F-SF by means of its relationship with the CFS-11. Finally, we evaluated the utility of the PROMIS-F-SF to identify patient-status by conducting receiver operating characteristic curves.

**Results:**

70 healthy volunteers and 62 patients completed the instruments. The PROMIS-F-SF had a average fit to the previously reported single-factor structure. Cronbach’s alpha and McDonald’s omega showed good internal reliability (α = 0.96, ωtotal = 0.97). PROMIS-F-SF score was positively correlated with the CFS-11 (*r* =.76) and it correlated highly with depression (*r* =.78) and anxiety (*r* =.74) score. The optimal cut-off point in the ROC-analyses was 15, which yielded a sensitivity of 89% and a specificity of 67% in the prediction of patient status.

**Conclusions:**

Level of fatigue among psychiatric outpatients is high in patients with psychiatric illness, compared to levels measured in healthy volunteers. The Danish PROMIS-F-SF shows good psychometric properties in this combined sample of healthy adults and psychiatric patients with non-psychotic disorders and it is recommended as PRO measure for psychiatric populations. Examination of psychometric properties in patient populations with somatic disorder could be a natural next step.

## Background

There is a lack of consensus in the definition of fatigue [1], but it is often described as extreme and persistent tiredness, weakness or exhaustion that can be mental, physical or both [1, 2]. Smets et al. (1995) [3] proposes that fatigue is a multidimensional construct, which relates to at least the following domains mental fatigue, cognitive fatigue, emotional fatigue, motivational fatigue, and physical fatigue.

Physical and mental fatigue affects all segments of the healthy population [4, 5], where the prevalence of fatigue has been reported to range from 7 to 45% [6]. Further, fatigue is a common symptom of both somatic and psychiatric illness. Among patients with chronic somatic conditions, heart failure, and cancer, the prevalence is estimated to be as high as 80-90% [7, 8].

Fatigue is a prevalent unspecific symptom in populations of somatic patients with malignant illness, neurological diseases, asthma, chronic hepatitis, diabetes, hypothyroidism, anemia, and among those who have been exposed to environmental toxins [1]. Further, it is a prevalent and unspecific symptom in populations of psychiatric patients with depression, anxiety, and emotional stress [9, 10]. Finally, disorders like Chronic Fatigue Syndrome, neurasthenia, functional somatic disorders, and sleep disorders have fatigue as a main symptom. Fatigue is, therefore, a transdiagnostic symptom that spans across somatic and psychiatric illnesses.

Fatigue is a subjective feeling and therefore relevant to assess as a patient-reported outcome (PRO). PRO is an umbrella term, which describes outcomes, collected directly from patients (in contrast to clinician reported outcomes). These are outcomes which are not subject to interpretation by clinicians or researchers and at the same time meaningful descriptors of the patients’ illness experience, suffering or coping [11].

A number of different instruments has been designed to assess fatigue. A recent review by Billones et al. (2021) [1] identified 27 different validated clinical measures used to assess fatigue and its dimensions. Examples are the eleven-item Chalder Fatigue Scale (CFS-11) [12] and the 30-item Multidimensional Fatigue Inventory (MFI) [3].

In 2004, the US National Institutes of Health (NIH) funded the Patient Reported Outcomes Measurement Information System (PROMIS), which was specifically developed as a standardized tool for measuring PROs [13]. It is a battery of PROs designed to assess a wide range of symptoms in both adult and pediatric populations. The items in PROMIS stem from other instruments (known as “legacy measures”) designed to assess the construct in question (e.g., fatigue). An advantage over other disease-specific PROs that capture responses regarding a specific condition is that PROMIS measures are applicable *irrespective of diagnosis*. PROMIS includes more than 300 person-centered measures of symptoms and functioning in the domains of physical, mental, and social health. Among them is the 95-item PROMIS Fatigue (PROMIS-F), which is part of the physical health domain of the larger Adult Self-Reported Health framework in the PROMIS.

The PROMIS-F also exist in a number of derivative short forms (4a, 6a, 8a, and 7a) with four to eight items (PROMIS-F-SF). The PROMIS-F-SF 7a has in research been found to be highly correlated with the PROMIS-F (*r* =.92) [14]. While other PROMIS-F-SFs has been validated in patients with fibromyalgia [15], human immunodeficiency virus (HIV) [16], multiple sclerosis [14], the 6a has only been subject to little research.

The PROMIS-F-SF 6a has been translated into Dutch-Flamish [17] and Danish [18]. The PROMIS-F-SF 6a has only been formally validated in populations of patients with endometriosis-associated pain [19]. Further, it has been validated in the healthy Dutch population [17].

The primary focus of this paper is the PROMIS-F-SF 6a. For the sake of brevity and clarity, it will be referred to as the PROMIS-F-SF throughout the paper.

No PROMIS-F-SF has not been psychometrically evaluated in populations with mental illness and has only, to a limited extent, been evaluated in non-American populations [17]. Therefore, in this cross-sectional analysis, we examine the level of fatigue in a Danish population of patients with anxiety, depression and personality disorders. We further examine the psychometric properties of the PROMIS F-SF, and test the agreement with a well-established longer instrument, the Chalder Fatigue Scale (CFS-11), which has been applied in Chronic Fatigue [20].

When collecting PRO from inpatients or other low-function populations and if collecting PRO frequently, i.e., weekly or bi-weekly, it is imperative for adherence to apply brief scales. Hence, for clinical and research applications, it is relevant to examine the psychometric properties of brief scale variants even though the full-length scales have been validated previously. Developing PRO instruments that embrace subjective distress types that are transdiagnostic regarding different types of mental disorders but also regarding most somatic disorders will strengthen research in the patient of tomorrow characterized by multi-morbidity [21].

Here, we report a validation of the Danish translation of the PROMIS-F-SF. The PROMIS-F-SF was administered to a population of patients receiving treatment for non-psychotic disorders and a population of healthy volunteers. We (1) conducted a confirmatory factor analysis of the Danish PROMIS-F-SF fit to the single-factor model; (2) examined the internal consistency reliability of the Danish PROMIS-F-SF; (3) evaluate the agreement between PROMIS-F-SF, the CFS-11, and single-items #15 and #20 of the BDI-II, which are related to fatigue in the Beck Depression Inventory-II [22]; and (4) assessed the level of fatigue in the psychiatric population with emotional disorders compared to the healthy volunteers.

We hypothesize that the Danish PROMIS-F-SF questionnaire will have a single-factor structure; good psychometric properties; a good agreement with the CFS-11 and with the fatigue-items of the BDI-II as well as the total BAI, and the BDI-II; that psychiatric patients will have higher levels of fatigue compared with healthy volunteers; and that the PROMISE-F-SF can discriminate patients from healthy volunteers.

## Methods

### Setting and procedure

Patients were recruited in outpatient secondary care clinics run by Region Zealand Mental Health Services (MHS). The patients found in these clinics are complex patients who have failed to respond to at least one line of treatment in the primary care sector. We advertised for respondents on posters in the waiting rooms of four clinics. These posters included information concerning the present study and a link to an online survey.

Healthy volunteers were recruited among staff of the Region Zealand MHS, who was given a link to the online survey. Other healthy volunteers were recruited through an online survey on social media.

### Online survey

Patients and healthy volunteers gave informed consent on the first page of the online survey. The online survey collected self-reported information regarding psychiatric diagnosis, age, and sex. It also included the Danish PROMIS-F-SF, the CFQ, the BDI-II, and the BAI. Data was collected between September 2021 and July 2022.

### Ethical considerations

The study was in accordance with local regulations registered with the Danish Data Protection Agency Region Zealand (REG-048-2021). The survey study did not, as per local guidelines and regulations, need approval by the Region Zealand Ethics Committee. Informed consent was taken from participants to participate in the study.

### Instruments

#### PROMIS Item Bank v1.0—Fatigue—Short Form 6a (PROMIS-F-SF)

The PROMIS F-SF 6a includes six items designed to assess the subjective feeling of fatigue and along with the interference of fatigue in daily life and activities. The instrument has a recall period of the last seven days. Examples of items include; “How often did you feel tired,” and “How often were you too tired to take a bath/shower?“. Each item is rated on a five-point Likert scale ranging from 1 = never to 5 = always. The questionnaire is reported as a total score, which is obtained by summing up the scores of all items. Scores can range from 6 to 30, with higher scores indicating greater fatigue. We used a translation of the instrument by researchers at the Section of Social Medicine, Department of Public Health, University of Copenhagen, which translated the PROMIS physical function item bank [18]. The Danish version can be obtained from the translators of the instrument.

#### The Chalder Fatigue Scale, eleven items (CFS-11)

The Chalder Fatigue Scale (CFS-11) is a questionnaire originally designed by the research team of Trudie Chalder at King’s College London to measure the severity of tiredness in fatiguing illnesses. The CFS was originally developed as a 14-item scale (CFS-14) in 1993 to assess perceived fatigue [23]. In 2010, the original instrument was revised, and a shorter instrument was published with three fewer items [12]. We applied this version of the CFS with 11 items. It has been found to have the following two subscales: physical fatigue (CFS-PF) and mental fatigue (CFS-MF) [12]. The items are either scored with a bimodal scoring system or with a Likert scale. We applied the Likert scoring. Here, each item is rated on a four-point Likert scale with the options: from 1 = Less than usual; 2 = No more than usual; 3 = More than usual; and 4 = Much more than usual. Sum-scores can range from 0 to 33 points, with higher scores indicating greater fatigue. Research has found the English version of the CFS-11 to have good psychometric properties [12]. We used a translation of the instrument previously used in Danish epidemiological research [24]. The questionnaire has not been formally validated in Danish.

#### The Beck Anxiety Index (BAI)

The Beck Anxiety Index (BAI) is a 21-item self-report instrument designed to assess the severity of anxiety in adolescents and adults ages 17 and older. The BAI asks about common symptoms of anxiety that the subject has had during the past week (including the day they take it). Each of the items is scored on a four-point Likert scale from 0 = Not At All to 3 = Severely—it bothered me a lot. Scores can range from 0 to 63 points, with higher total scores indicating more severe symptoms of anxiety [25]. We used a Danish translation published by Pearson © [26].

#### The Beck Depression Inventory, second edition (BDI-II)

The Beck Depression Index, second edition (BDI-II) is a 21-item self-report instrument designed to assess the severity of depression in adolescents and adults ages 17 and older. The BDI-II asks about common symptoms of depression that the subject has had during the past two weeks (including the day they take it). It is the second revision of the original Beck Depression Index, which was published in 1961 [27]. The second version was published in 1996 and was developed in response to the American Psychiatric Association’s publication of the Diagnostic and Statistical Manual of Mental Disorders, Fourth Edition, which changed many of the diagnostic criteria for Major Depressive Disorder. Each of the items is scored on a four-point scale from 0 to 3. Scores can range from 0 to 63 points, with higher total scores indicating more severe depressive symptoms [28]. The BDI-II includes #15 “Loss of Energy” and #20 “Tiredness or Fatigue” which both relates to fatigue. We used a Danish translation published by Pearson © [29].

### Statistical analyses

We undertook all data processing and analyses using R 4.3.0 (Already Tomorrow) and RStudio 2022.07.2 + 576 [30], including the psych 2.1.9 [31], lavaan 0.6–9 [32], and cutpointr [33] R packages.

First, we report descriptive statistics. We transformed the simple sum scores score on the PROMIS-F into T-scores, by manually converting each score into a T-score from the table provided in the PROMIS-F-SF manual [34]. We examined for ceiling and floor effects. It is important to explore ceiling and floor effects, as such can indicate that an instrument might be insensitive to change or population differences. Ceiling effects were explored based on the highest response option, and floor effects were based on the lowest response option. Proportions ≥ 15% of people at either end were considered evidence of an effect [35]. We evaluated ceiling and floor effects on a scale level as suggested by Mchorney and Tarlov (1995) [35].

Secondly, we carried out confirmatory factor analyses (CFA) of the Danish PROMIS-F-SF to evaluate its fit to the proposed single-factor model. We used the lavaan R [32] package with the MLM estimator and treated data as continuous. We first carried out single-group analysis and then carried out multigroup analysis with 2 subgroups (patient status yes or no). We calculated, as recommended by Kline (2015), the comparative fit index (CFI), the Tucker-Lewis index (TLI), the root mean square error of approximation (RMSEA), the standardized root mean square residual (SRMR), and the degrees of freedom (df) [36]. We utilized the criteria set forth by Hu and Bentler, which suggest that an RMSEA smaller than 0.06, an SRMR smaller than 0.08, and a CFI and TLI larger than 0.95 indicate relatively good model—data fit [37]. The chi-square fit statistic is usually evaluated as the ratio of the chi-square statistic to the respective degrees of freedom (χ2 /pdf) [38]. A ratio of smaller than 2.6 indicates a superior fit data [39].

Third, to evaluate internal consistency reliability of the Danish PROMISF-SF we calculated Cronbach’s alpha (α), McDonald’s hierarchical omega (ωh), and the total omega (ωtotal). α above 0.70 was considered satisfactory [40, 41], and so were ωh above 0.65 and ωtotal above 0.80 [42].

Fourth, to evaluate the convergent validity of the Danish PROMIS-F-SF, we calculated its Pearson’s correlation with a legacy measure of fatigue, the CFS-11 total score. We also evaluated to which degree the Danish PROMIS-F-SF correlated with single items #15 and #20 of the BDI-II, which concerns fatigue. We also calculated the correlation between the PROMIS-F-SF, the BAI total score, and the BDI-II total score. Correlations less than 0.30 were considered weak, correlations between 0.30 and 0.49 were considered moderate, and correlations greater than 0.49 were considered strong [43].

Fifth, we evaluate the difference in mean scores on the PROMIS-F-SF and the CFS-11 between populations using Welch’s t-tests.

Lastly, receiver operating characteristic (ROC) curves were made, and the Area Under the Curve (AUC) was calculated, testing sensitivity for patient status, which was defined as the patient reporting affiliation with mental health services. We utilized the “cutpoint” function to calculate the cut-off point, which had the best overall sensitivity and specificity. We regarded an AUC above 0.9 as excellent, > 0.8 as good, > 0.7 as fair, and < 0.7 as poor [44].

## Results

### Descriptive statistics

One hundred and thirty-two individuals were recruited for the study. Of these, four individuals with missing data were excluded. Of the resulting 128 respondents, 62 were patients and 66 were healthy volunteers. The majority of the sample was young (39.5 years, SD = 11.65) and female (82.0%). Most of the patients suffered from anxiety disorders (30.2%). See Table 1 for characteristics of the included healthy volunteers and patients. Healthy volunteers had a mean PROMIS-F-SF t-score of 49.92 [8.41] and patients with Borderline PD had the highest score of 64.74 [7.40]. See Table 2 for PROMIS-F-SF scores for the total sample, healthy volunteers, and patients.

**Table 1:** Descriptive statistics

	Total sample [SD] *N* = 128	Patients [SD] *N* = 62	Healthy volunteers [SD] *N* = 66
Female	105 (82.0%)	53 (41.4%)	52 (40.6%)
Age (mean) [standard deviation]	39.5 [11.65]	37.65 [11.99]	42.83 [10.81]
Diagnosis			
Borderline PD		17 (27.4%)	
Anxiety disorders		19 (30.2%)	
Depression		16 (25.8%)	
Other		11 (17.5%)	

**Table 2:** PROMIS-F-SF scores

	*N*	T-score Mean [SD]	T-score range	Sum score [SD]	Range
**Total sample**	128	55.64 [10.24]	43.4	17.01 [6.74]	24
Healthy volunteers	66	49.92 [8.41]	43.4	13.06 [5.07]	24
Patient population	62	61.74 [8.36]	43.4	21.22 [5.70]	30
**Diagnosis**					
Borderline PD	17	64.74 [7.4]	27.4	23.31 [5.22]	18
Anxiety disorders	19	63.77 [8.23]	29.0	22.42 [5.56]	19
Depression	16	58.71 [9.26]	37.6	19.38 [6.03]	22
Other	11	58.26 [6.6]	20	18.73 [5.0]	15

[] are used to indicate standard deviations

There was a difference in PROMIS-F-SF score between patients and healthy adults, where patients had higher scores (t = 8.529, df = 122.04, p = > 0.01). There was also a difference in PROMIS-F-SF score between patients with borderline PD and patients with another diagnosis, where patients with borderline PD had higher scores (t = 2.2983, df = 22.274, *p* =.03), but not for depression or anxiety.

### Ceiling and floor effects

4.7% of the included patients scored the lowest possible score and 3.9% scored the highest possible score. The scale shows no evidence of floor or ceiling effect.

### Factor structure

#### Single-group analysis

Single-group analysis CFA showed a poor to average fit to the single-factor model previously suggested in the literature on most fit-indices (CFI = 0.977 TLI = 0.961, RMSEA [CI] = 0.133 [0.081, 0.188], SRMR = 0.022, Df = 9 and Chi2 = 29.276) All the factor loadings were significant and indicated good loading of the items onto the single factor (range: 0.963 to 1.106). However, the chi-square/df value for the single-factor model was 3.25, which should have been below 2.6.

#### Multi-group analysis

Multi-group analysis CFA improved the overall fit to the single factor model, and all fit indexes was good, except the RMSEA which was unsatisfactory. The result of the CFA showed an average to good fit to the single-factor model on most fit-indices (CFI = 967. TLI = 0.944, RMSEA [CI] = 0.139 [0.081, 0.196], SRMR = 0.032, Df = 18 and Chi2 = 40.136) All the factor loadings were significant across the two groups and indicated good loading of the items onto the single factor (range: 0.824 to 1.0 for patients and range: 1.0 to 1.252 for healthy volunteers). The chi-square/df value for the single-factor model was 2.23 and therefore below 2.6, and the RMSEA of 0.139 was still well above the 0.06 limit and was not improved.

### Reliability of the Danish PROMIS-F-SF

Internal consistency reliability was found to be good for the Danish PROMIS-F-SF (α = 0.96, 95% CI [0.95, 0.97]; ωh = 0.92, ωtotal = 0.97) for the entire sample. Subgroup-analysis also showed good internal consistency reliability for healthy volunteers (α = 0.94, 95% CI [0.92, 0.96]; ωh = 0.9, ωtotal = 0.97) and for the patient sample (α = 0.94, 95% CI [0.91, 0.96]; ωh = 0.9, ωtotal = 0.95).

### Convergent validity

#### Total sample

PROMIS-F-SF correlated strongly with the CFS-11 (r =.76, 95% CI [.68,.83], p = <.001). The PROMIS-F-SF correlated strongly with BDI-II (r =.78, 95% CI [.71,.84], p = <.001) and BAI (r =.74, 95% CI [.65,.81], p = <.001). It also correlated strongly with single items from the BDI #15 “Loss of Energy” (r =.73, 95% CI [.63,.80], p = <.001) and #20 “Tiredness or fatigue” (r =.78, 95% CI [.71,.84], p = <.001).

#### Healthy volunteers

PROMIS-F-SF correlated strongly with the CFS-11 (r =.64, 95% CI [.47,.76], p = <.001). The PROMIS-F-SF correlated strongly with BDI-II (r =.70, 95% CI [.55,.81] p = <.001) and BAI (r =.51, 95% CI [.32,.68] p = <.001). It also correlated strongly with single items from the BDI #15 “Loss of Energy” (r =.73 95% CI [.63,.80], p = <.001) and #20 “Tiredness or fatigue” (r =.70, 95% CI [.55,.81], p = <.001).

#### Patients

PROMIS-F-SF correlated strongly with the CFS-11 (r =.71, 95% CI [.55,.82], p = <.001). The PROMIS-F-SF correlated strongly with BDI-II (r =.66, 95% CI [.49,.79] p = <.001) and BAI (r =.62, 95% CI [.43,.75] p = <.001). It also correlated strongly with single items from the BDI #15 “Loss of Energy” (r =.65 95% CI [.47,.77], p = <.001) and #20 “Tiredness or fatigue” (r =.71, 95% CI [.55,.81], p = <.001).

### The external validity of the PROMIS-F-SF

**Fig. 1:**
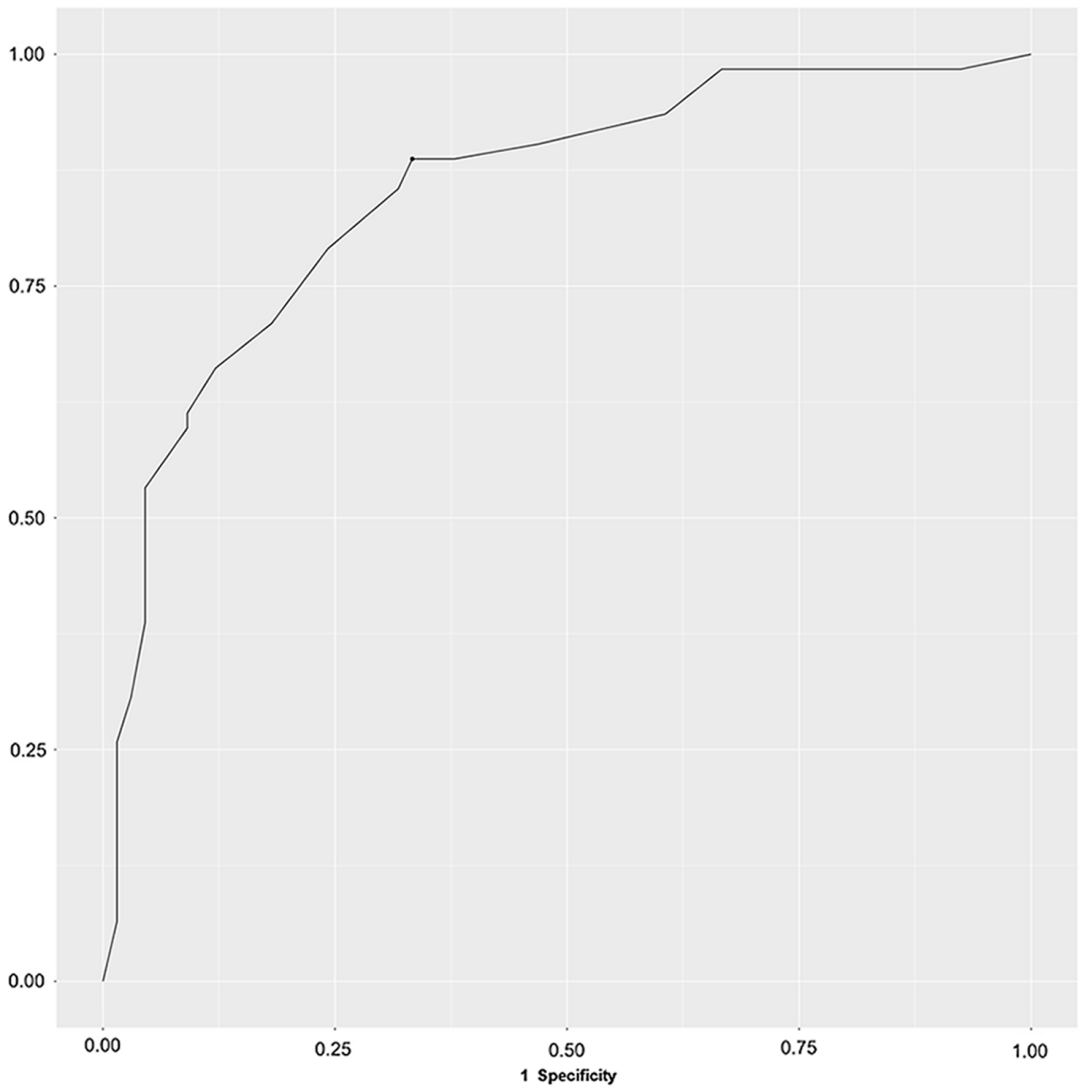
ROC Curve testing for sensitivity for patient status

ROC curves testing for sensitivity for patient status (*N* = 128) are presented in Fig. 1. The optimal cut-off point across diagnostic groups was found to be 53.7 and yielded a sensitivity of 89% and a specificity of 67% with a good accuracy as indicated by an AUC = 0.85.

## Discussion

This is, to the best of our knowledge, the first time the PROMIS-F-SF has been applied in any psychiatric sample and only the second time it has been applied in a non-English version. We found that the population of patients with emotional disorders reported a significantly greater level of fatigue than the healthy volunteers. The individuals with a diagnosis of borderline personality disorder reported the highest level of fatigue, and fatigue was correlated with levels of self-reported anxiety and depression. The fatigue reported by patients with borderline PD was marginally smaller than the level of fatigue reported in similar research which has applied the PROMIS-F-SF in populations of patients with endometriosis-associated pain (T-score 63.3) [19]. Our healthy sample had levels of fatigue almost identical to Dutch healthy adults (T-score 49.1) [17].

PROMIS-F-SF had good internal consistency reliability, which corresponds with the finding of researchers who have applied the original PROMIS-F-SF in outpatients with endometriosis-associated pain [19], sickle cell disease [15], and cardio metabolic risk [15] (alpha of 0.93, 0.88, and 0.86 respectively). We have found no studies, which publishes omega values.

The PROMIS-F-SF correlated to a high degree with the longer legacy instrument CFS-11. This suggests that the PROMIS-F-SF successfully captures the same construct as the legacy instrument but does so with fewer items. In retrospect, is it possible we instead should have compared the PROMIS-F-SF to another legacy instrument, the Multidimensional Fatigue Inventory (MFI) [3], as it also assesses motivational, emotional, and cognitive domains of fatigue [1], while the CFS-11 only assessed mental and physical fatigue [1]. It is, as such, possible that the PROMIS-F-SF fails to capture essential domains of fatigue, which the CFS-11 also fails to capture.

The PROMIS-F-SF also correlated to a high degree with single items about fatigue from the BDI-II, and it is, therefore, questionable whether the PROMIS-F-SF provides extra information about fatigue if BDI-II is also collected. While BDI-II was constructed for application in a patient group with depression, it is frequently applied as screening instrument and as a transdiagnostic measure of depressive symptomatology across diagnostic categories. BDI-II contains 21 items and as such is too long for use as a frequently repeated PRO instrument. Whether it is feasible to apply selected items (i.e. #15 and #20) as PRO tool should be investigated in more detail, in a population with depression and across a range of diagnoses.

We found no evidence of ceiling-effect on the Danish PROMIS-F-SF. This indicates that the instrument is suitable to monitor fatigue even aggravation in populations with high baseline fatigue level (e.g., following a medical intervention, which could increase fatigue).

We analyzed the factor structure of the Danish PROMIS-F-SF with confirmatory factor analysis methods. We found that the Danish translation had an average to good fit with the single factor model suggested for PROMIS-F-SF in previous confirmatory factor analyses [15, 19] on most fit-indices, but not on the RMSEA and the chi-square/df value. A possible explanation for the less-than-optimal fit on some fit indices could be that the PROMIS-F-SF is a heterogeneous index made from many different items, which in the literature has been associated with fatigue, and not a homogeneous domain.

Lastly, we examined the PROMIS-F-SF specificity and sensitivity for detecting patient status and found good abilities to do so. This suggest that elevated fatigue level is a phenomenon, which only to a limited degree is observed in the healthy population. Other psychometric studies of the PROMIS-S-SF have not reported data on the instrument’s external validity regarding patient status.

A number of limitations of the present study has to be addressed. First, this validation of the PROMIS-F-SF was obtained from patients who found the survey link in a psychiatric outpatient clinic and who self-reported their diagnosis. It is possible that some of these patients have not yet undergone formal diagnostic assessment in the clinic; instead, they report what they identify as the best description of their psychological difficulty. Considering this, is it possible that the fatigue-levels might not be specific to the mentioned diagnostic categories. Relating to this, the patients was recruited from secondary care outpatient clinics, which receives treatment-resistant patients from the primary care sector. Therefore, might the included patients represent more severe cases of their respective mental health conditions, which might limit current study in terms of applicability of the findings for less severe patient populations.

Further, the healthy volunteers were recruited both by spreading the link on social media and among colleagues in the Region of New Zealand Mental Health Services. It is, therefore, possible that some of the healthy volunteers have under-reported some or all-psychiatric symptoms. Secondly, results are based on data from relatively few subjects.

These limitations could be addressed in future research on the Danish PROMIS-F-SF, which can be conducted in patient populations with other psychiatric disorders or somatic illnesses. This can further enrich the understanding of the PROMIS-F-SFs abilities to rapidly quantify fatigue and support the use of it in future routine quality-assurance data collection and in clinical trials.

In conclusion, the findings of the present study indicate that patients with personality disorder, anxiety or depression all have elevated levels of fatigue comparable to patients with endometriosis and clearly higher than healthy subjects. The Danish PROMIS-F-SF has the same good internal validity as the original English scale, and it is adequately described by the proposed single-factor structure and has good sensitivity and specificity in identifying patient populations.

## Data Availability

The datasets used and analyzed during the current study are available from the last author on reasonable request.
